# Can new urbanization pilot policies promote green technology innovation in cities: Empirical evidence from China

**DOI:** 10.1371/journal.pone.0303404

**Published:** 2024-05-07

**Authors:** Jing Cheng, Jiarui Chen

**Affiliations:** School of Humanity & Social Science, Jiangsu University of Science and Technology, Zhenjiang, China; IBS Hyderabad: ICFAI Business School, INDIA

## Abstract

The development of urbanization has brought new challenges to the ecological environment, and the promotion of green technology innovation and development is widely recognized as an essential method to achieve cities’ economic benefits and environmental protection. This paper examines whether the new urbanization pilot policies (NUP) increase green technology innovation (GTI) from both theoretical and empirical perspectives. This paper examines the impact of new urbanization on GTI by analyzing data from 285 cities in China between 2010 and 2021, using the multi-period DID model with the implementation of NUP as an exogenous policy shock. The study results indicate that NUP significantly affects GTI, and the conclusion still holds after the parallel trend test, placebo test, and other robustness tests. Heterogeneity analysis shows that the NUP significantly enhances GTI in low environmental pollution, non-resource-based, Medium-sized, and Central Region cities. The test of moderating effect shows that NUP has a "linkage effect" with the government’s environmental attention, financial investment in innovation, and regional talent pooling. The findings of this paper provide empirical evidence and decision-making reference for promoting NUP and sustainable development of cities.

## Introduction

Exploring sustainable sources of economic growth has always been a critical topic in economics research. During the initial phase of China’s urbanization and industrialization, the surplus of labor resulting from the dual economic and social structures of urban and rural areas, along with the heavy industry development strategy relying heavily on resources like coal and minerals, significantly contributed to China’s economic growth. However, with the emergence of "Lewis turning point" characteristics in China, the sustainability of the labor-dependent growth model is being increasingly questioned [1]. The outdated economic development model, characterized by high resource consumption, environmental pollution, and low economic efficiency, is causing many cities to confront environmental pollution and urban decline simultaneously [2]. In this context, changing the mode of economic development and promoting the economy from the high-speed development stage to the high-quality development stage have become necessary for China’s current and future economic development.

As the first driving force for development, innovation is the core and essential support for promoting green development and building a beautiful China. GTI, as a development strategy integrating innovation drive and green development, is conducive to realizing a "win-win" situation for urban economic benefits and environmental protection and is a critical way to solve the dilemma of urban pollution and decline [3, 4]. The China’s government work report released in 2022 proposes to "Promote the research and development and popularization and application of green and low-carbon technologies, accelerate the formation of a green production and living style, deal with the relationship between development and emission reduction, promote the low-carbon energy transition, and implement the Carbon Peak Action Plan". Furthermore, the State Council of the PRC in 2023 issued a white paper on "China’s Green Development in the New Era", which further emphasized that "Take scientific and technological innovation as the driving force and guarantee for adjusting the industrial structure and promoting the green and low-carbon transformation of the economy and society". A comprehensive examination of the strategies to bolster GTI is of paramount importance, offering profound theoretical insights and policy guidance. This study aims to delve into the role of NUP in enhancing GTI, assessing their efficacy in fostering a sustainable and environmentally responsible economic trajectory. By integrating empirical evidence with theoretical frameworks, this research endeavors to provide a nuanced understanding of the dynamics between urbanization, GTI, and sustainable development, thereby contributing to the policy discourse on sustainable urban growth and ecological innovation in China.

## Literature review

Regarding the factors influencing the level of GTI, scholars have carried out research from different perspectives. Among them, the most discussed issue is how government intervention affects GTI. The Porter hypothesis suggests that, if properly designed, environmental regulation (especially market-based instruments) can achieve "innovation compensation" [5]. As a means for governments to intervene in economic development, environmental regulation can incentivize firms to promote green innovation by imposing environmental constraints while improving the ecological environment [6]. Firms that maintain a high level of green innovation and the number of green patents are more likely to stay ahead of the curve and maintain market share [7]. However, some scholars have also argued that government subsidies crowd out the share of investment that firms would otherwise spend on R&D, which to some extent is detrimental to firms’ GTI [8], and that environmental regulations may even inhibit the efficiency of technological innovations and reduce productivity in the manufacturing sector [9]. In addition, economic growth pressures may also distort government decisions on environmental regulation and GTI [10]. In regions with higher economic growth pressures, the government tends to focus resources on developing its advantageous industries while also lowering regional environmental regulations to bring in foreign investment, which can have a certain crowding-out effect on GTI [11]. In addition to environmental regulations and subsidies for technological innovation, the implementation of different pilot policies by the government is also crucial in influencing the level of GTI in the region, e.g., the policy of ecological civilization demonstration zones [12], the pilot policy of carbon emissions trading [13], and the policy of environmental information disclosure [14]. In addition to the important factor of government intervention, factors such as industrial robotics [15], digital finance [16], institutional quality [17], and green finance [18] also affect the level of GTI.

The above research results help to deepen the understanding of the factors of local GTI. As an innovation to the traditional urbanization development model, NUP is an important initiative to promote sustainable regional development in China. However, few studies have explored the impact of NUP on local GTI from the perspective of pilot policies. The first is the environmental improvement role of NUP, which is similar to the research in this paper but with a different focus. Existing studies have shown that implementing NUP effectively reduces pollution emissions and improves energy efficiency [19], and significantly improves urban air quality [20]. Li et al. (2023) [21] argue that NUP belongs to the institutional level, advocating the integration of ecological civilization and urban development, aiming to deal with the various ecological problems and development quality problems arising in the context of China’s large-scale urbanization development. Zhang et al. (2023) [22] take enterprises as their research samples and argue that NUP is a kind of "soft constraint" for enterprises, which can significantly increase the green technology level of enterprises in the pilot cities. Second, the rural development aspect of NUP. Under the guidance of the two strategies of new urbanization and rural revitalization, the factors between urban and rural areas have changed from "one-way flow" to "bilateral interaction", and China’s urban and rural development policies have changed from "urban bias" to "bilateral interaction" [23]. Specifically, the NUP can alleviate the pressure brought about by the over-concentration of large cities by promoting the local urbanization of rural residents and focusing on the absorption of rural employment by small towns in order to promote balanced regional development and balanced development of urban and rural areas [24], and the income gap between urban and rural areas can be further narrowed [25].

Based on the above analysis, this paper focuses on answering the following questions: Whether the new urbanization development model will enhance the level of GTI in cities? Will the environmental pollution problems of cities be alleviated due to the NUP’s realizing the high-quality development of the regional economy? Does the implementation of NUP have a heterogeneous impact on cities with different characteristics?

The establishment of the first batch of new urbanization pilot projects in China in 2014, which can be regarded as a natural experiment of the new urban development model, provides an excellent opportunity for this paper to answer the above questions. In the context of the deep development of urbanization, the study of the impact of a new type of urbanization development model has significant theoretical value and practical guidance significance.

Compared with the existing literature, the possible contributions of this paper are: (1) This paper contributes to the literature by theoretically expanding the understanding of how government behavior influences GTI. It posits that government actions through NUP are not merely direct stimulators of GTI but also function through indirect pathways, such as environmental regulation, public service provision, and talent aggregation, which collectively create an ecosystem conducive to innovation. (2) We innovatively applies Porter hypothesis to the context of urbanization, suggesting that appropriate environmental regulations within the framework of NUP can lead to increased innovation activities among firms. This theoretical integration provides a new perspective on how environmental policies can drive economic growth through enhanced GTI. (3) By applying agglomeration theory to the GTI context, the paper offers a theoretical explanation for how population and industry agglomeration can lead to economies of scale and improved GTI. This theoretical application provides insights into the spatial dynamics of innovation and economic growth. (4) In terms of conceptualization of GTI development system, we present a conceptual framework of a green innovation development system under NUP, which includes policy preferences, financial support, and continuous supervision. This theoretical construct provides a new perspective to understanding the multifaceted role of government in nurturing GTI within urban settings.

The remainder of the paper is organized as follows: The second part is the policy background and theoretical analysis. The third part is the research design, including variable definition, model design, and descriptive statistics. The fourth part is the empirical analysis, including the benchmark test and robustness test. The fifth part is further analysis, including tests of moderator effects and heterogeneity analysis. The sixth part is the conclusion and policy recommendations.

## Policy background and research hypothesis

### Policy background

Against the backdrop of rapid industrialization and the migration of large numbers of agricultural populations from the countryside to the cities, China is promoting urbanization despite objective contradictions including inadequate provision of essential public services, relative scarcity of urban resources, a fragile ecological environment, and an imbalance between urban and rural development, among others. In March 2014, The State Council of the PRC issued the National Plan for New-Type Urbanization (2014–2020), the overall objective of which is to improve the overall quality and level of urbanization by clarifying the path of urbanization development, primary objectives and strategic tasks.

In order to implement the requirements of the National New Urbanization Plan (2014–2020), the National Development and Reform Commission(NDRC) and other 11 departments issued the Circular on the Comprehensive Pilot Work of National New Urbanization in 2014, listing Jiangsu and Anhui provinces and 62 cities (towns) such as Ningbo as the first batch of comprehensive pilot areas for national new urbanization, and required to start piloting by the end of 2014; in November 2015, the principle of giving priority to small and medium-sized cities, counties, established towns, and qualified development zones, as well as national new areas with a strong willingness to reform, high development potential, and concrete measures, resulted in the listing of 59 cities (towns), including Fangshan District of Beijing and other cities (towns), as the second batch of national pilot areas for new urbanization; and in December 2016, 111 cities (towns) were further selected to be listed as the third batch of national pilot areas for new urbanization. In order to ensure that the pilot cities seize the opportunity to promote the work related to new urbanization, NDRC will strengthen the tracking and supervision of the pilot work, carry out annual assessments and evaluations, and establish a mechanism for dynamic elimination of the pilots. The pilot areas shall summarize the pilot experience promptly, ensure the completion of the tasks and objectives of the comprehensive national pilot project on new urbanization, achieve the milestone results of the pilot tasks two years after the launch of the pilot project, and form replicable and generalizable experiences, and promote the successful experiences of the pilot project in an orderly manner nationwide in 2020.

**Fig 1:**
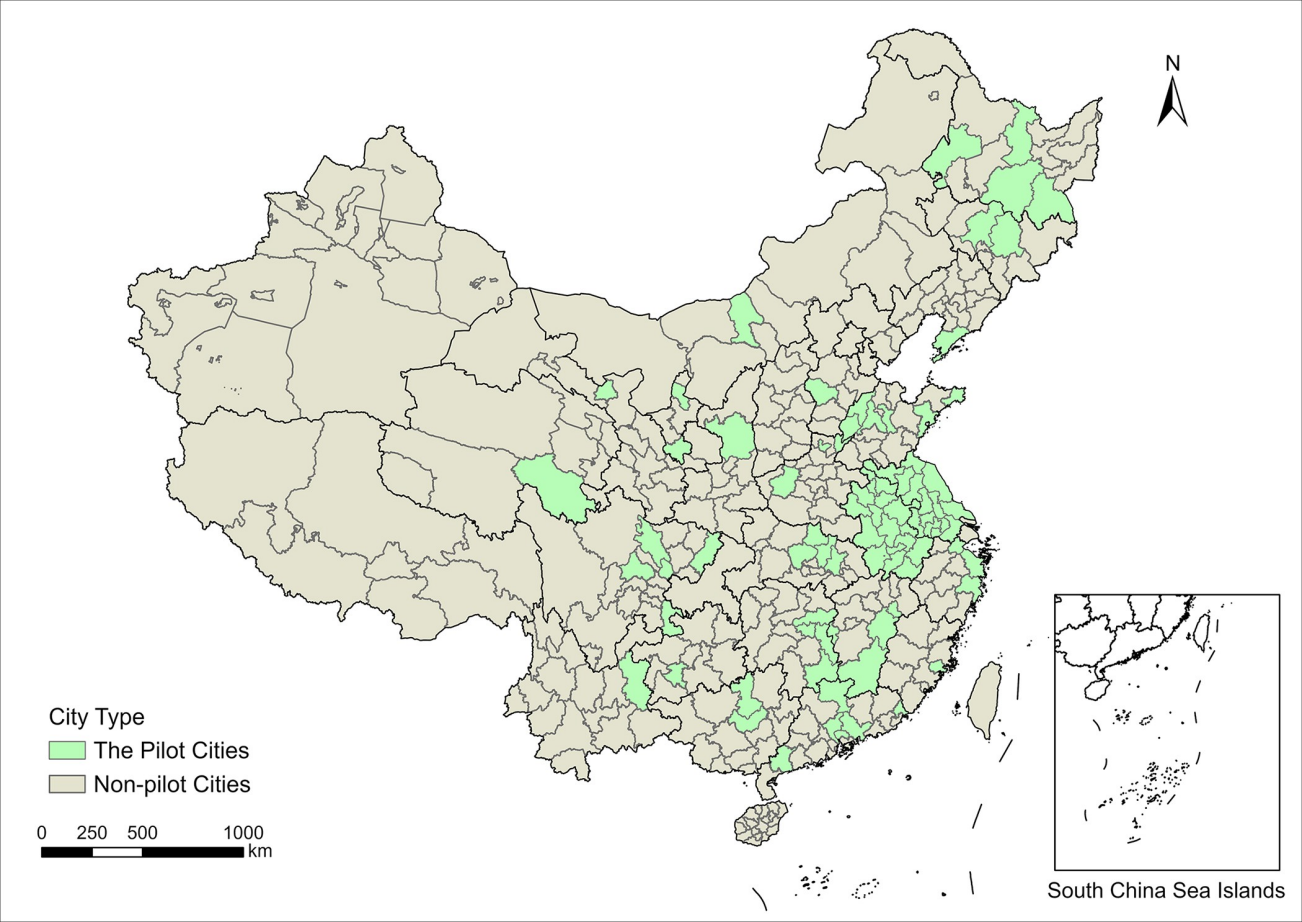
Spatial distribution of the pilot cities and non-pilot cities in China. Base map obtained from Tianditu (www.tianditu.gov.cn).

Improving cities’ capacity for sustainable development has always been a crucial component of building modern urbanization, whether it is found in the 14th Five-Year Plan’s national implementation program for new urbanization or the three pilot programs for new urbanization. The NUP clearly states that it is necessary to advocate institutional mechanisms to promote green, circular, and low-carbon development of urbanization, implement the most stringent ecological environmental protection system, and form a spatial pattern, industrial structure, mode of production, and way of life that saves resources and protects the environment. On the other hand, it is essential to conform to the emerging tendencies of scientific and technological advancement as well as the transformation of the industrial sector, to give full play to the role of urban innovation carriers, to rely on the benefits of science and technology, education, and human resources, and to encourage cities to follow the path of innovation-driven development. Considering the availability of sample data, we finally chose 83 prefecture-level cities as the sample of the treatment group. Fig 1 shows the distribution of pilot cities and non-pilot cities.

### Theoretical analysis and research hypothesis

The NUP guides the reasonable concentration of industry and population through reasonable policy intervention and market competition mechanism, induces technological innovation, overcomes the negative market externalities through government regulation, and ultimately generates economies of scale to improve green technological innovation. Aghion et al. (2021) [26] conceptualize "creative destruction" as a dynamic process where the old contends with the new, with the growth effects of innovation being progressively actualized through government protection and support. The development of GTI in Chinese cities can also be regarded as a "creative destruction process," and government support is indispensable from the basic innovation stage to the stage of large-scale application of innovation. The crux of NUP is to bolster systemic top-level design, adhere to market principles, and advance reforms in pivotal areas and critical junctures, such as the ecological environment, to establish an institutional framework that supports the healthy progression of urbanization. Local governments under the NUP will implement a sustainable urbanization strategy and transform into investment-oriented governments responsible for the city’s GTI. From the stage of basic GTI to the stage of accelerated and large-scale application of GTI, the government under NUP can build a development system for the city’s green innovation through policy preferences, financial support, and continuous supervision, in which the government, the market, and the society can build, govern and share, and in which a variety of governance mechanisms can be complementary and embedded, to promote city’s GTI. Based on this, this paper puts forward the first research hypothesis:

**Hypothesis 1.** The NUP can promote the development of GTI in cities.

The Porter hypothesis suggests that appropriate environmental regulation can induce firms to engage in more innovative activities and that these innovations will increase firms’ productivity, thereby offsetting environmental protection costs and increasing firms’ profitability in the marketplace. Without government intervention, market forces do not usually promote a shift to GTI in a region [27]. It is mentioned in the Plan that the NUP should improve the mechanism of green, circular and low-carbon development of urbanization, establish an ecological civilization assessment mechanism, implement an ecological compensation system, establish a mechanism for trading resource and environmental property rights, and implement a stringent environmental regulatory system. As GTI has certain risks and uncertainties, and enterprises often face financial constraints when carrying out innovation, the government will appropriately increase the investment of government innovation funds while implementing environmental regulations to reduce the cost of technological innovation for enterprises [28, 29]. Therefore, when pollution emissions, environmental damage, and resource consumption in the pilot region are strictly regulated and restricted, environmental pollution by local firms will impose environmental regulation penalties, increasing firms’ production costs. Amidst the internal pressure of rising production costs and the external incentives of government innovation subsidies, firms are likely to ramp up their research and development (R&D) investments. This leads to an enhancement of their innovation capabilities, optimization of green technologies, and acceleration of product innovation. Consequently, governments can expect a reduction in regulatory costs, while firms can harness GTI to generate additional economic benefits. Based on this, this paper proposes the second research hypothesis:

**Hypothesis 2.** The impact of NUP on GTI may be moderated by government environmental attention and investment in innovation.

Classical theory of economic growth holds that total factor productivity increases depend on the free movement of factors of production, especially the free movement of labor. According to Salam et al. (2019) [30] and Abbass et al. (2022) [31], technical innovation and adoption drive economic growth through human capital and skill. In the actual development of China’s urbanization, the implementation of the rural land transfer policy liberated a large number of laborers to flock to the cities, but the restriction of the dual urban-rural household registration system prevented many migrants from integrating into the cities. However, this phenomenon has been improved after the implementation of the new urbanization policy. The new type of urbanization is not the same as the population transfer to the cities, but it emphasizes the human being at its core. The Plan states that "we must steadily push forward the coverage of the entire resident population with basic public services in urban areas, such as compulsory education, employment services, basic old-age pension, basic medical care and health care, and guaranteed housing". The key objectives of the NUP are to accelerate the reform of the household registration system, speed up the demobilization of the agricultural transfer population, enhance the ability to equalize basic public services, narrow the gap in the urbanization rate between cities, and achieve high-quality economic development. On the one hand, government spending more on new technologies helps to build an eco-friendly mechanism and helps to attain a green environment and achieve economic sustainability [32]. On the other hand, the new type of urbanization process is accompanied by factor mobility, industrial agglomeration, and improved transportation infrastructure construction, which will help cultivate high-quality talents and attract the agglomeration of high-skilled talents. According to the theory of agglomeration, the scale economy effect of the city will be played when the population agglomeration reaches the optimal scale, and the agglomeration of the population is conducive to the concentration of capital and labor and other factor resources, forming a "labor pool" [33], which will improve technology absorptive capacity [34] and green technology innovation capacity of cities in China. Based on this, this paper puts forward the third research hypothesis:

**Hypothesis 3.** The impact of NUP on GTI may be moderated by human capital.

**Fig 2:**
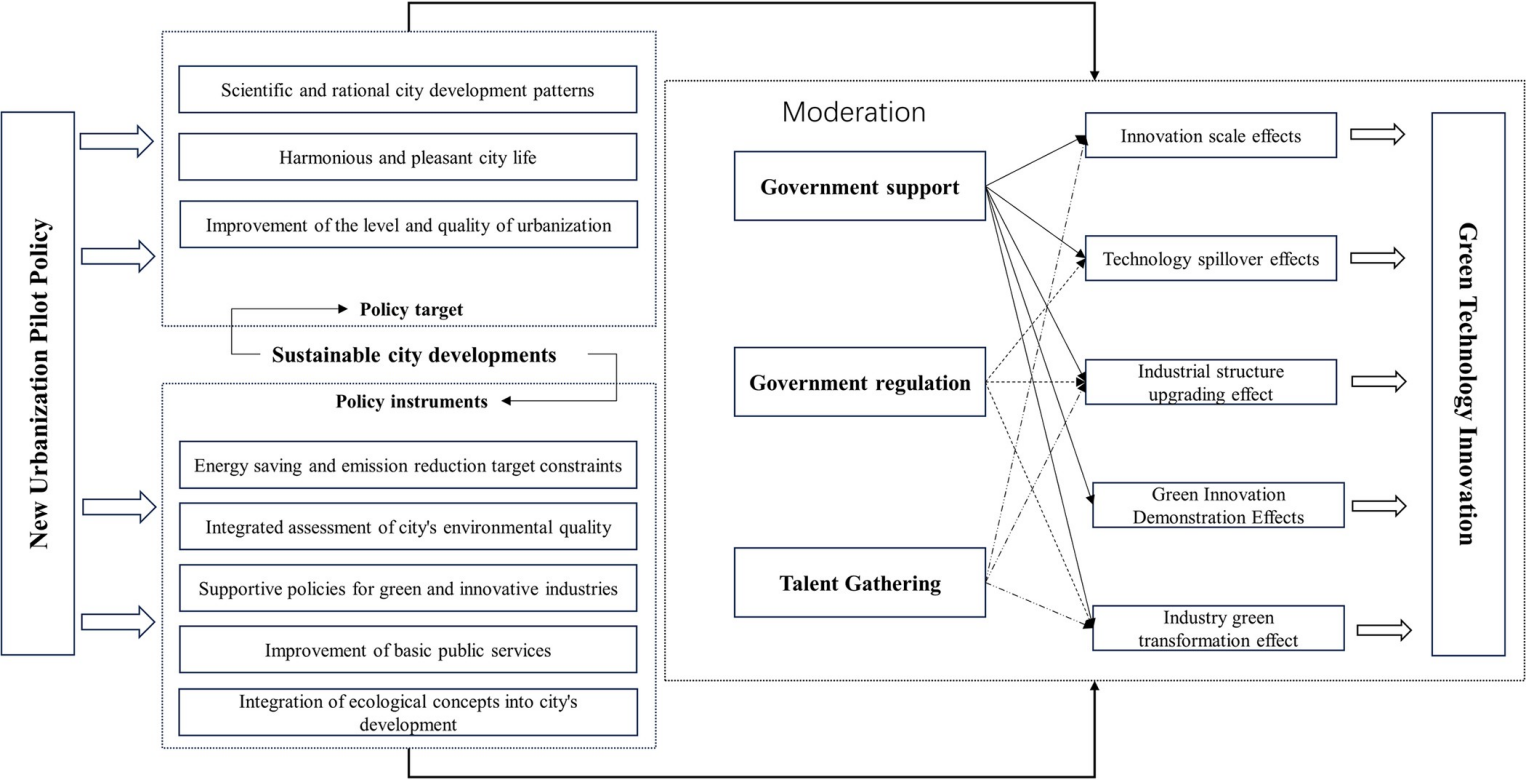
Theoretical framework of NUP promoting GTI.

Fig 2 illustrates the specific theoretical framework NUP contributes to GTI.

## Research design

### Model setting

#### DID model

Considering that the NUP has the characteristics of the first pilot and gradual roll-out, different batches of pilot cities start the pilot at different times. Therefore, this paper uses the multi-period difference-in-difference model (DID) to assess the causal relationship between NUP and GTI with the following benchmark model:

$$GTI_{it}=\alpha +\beta Treat_{i}\times Post_{it}+\gamma X_{it}+u_{i}+v_{i}+\varepsilon_{it}$$ (1)

The coefficient β is the core parameter estimated in this paper, indicating the impact of NUP implementation on the GTI level in cities. The subscript i represents city and t represents time. *GTI*_*it*_ is the level of GTI of city i in period t. *Treat*_*i*_ denotes a dummy variable for whether or not the area is the location for the NUP. *Post*_*it*_ denotes a point-in-time dummy variable about the implementation of NUP. *Treat*_*i*_×*Post*_*it*_ is the dummy variable used to identify whether the NUP has been implemented in city i at time t. *X*_*it*_ represents a series of control variables. *u*_*i*_ and *v*_*i*_ represent city-fixed effects and year-fixed effects, respectively. *ε*_*it*_ represents a randomized disturbance term and is clustered at the city level.

### Variable selection

#### Explained variable

The explanatory variable of this paper is the level of GTI, referring to the studies of Feng et al. (2022) [35]and Behera and Sethi (2022) [36], using the number of green invention patents obtained by the city in the year (in 10,000) to measure the status of GTI development in the city. Green patents are a common indicator of GTI. Existing studies have often used the number of green invention patent applications to measure the quality of green technological innovation and the number of green utility model patent applications to measure the quantity of GTI. Specifically, green invention patent applications must meet the requirements of novelty, creativity and utility, and they contain more independent intellectual property rights, which is a substantive green innovation achievement and, therefore, can reflect the level of GTI in cities. In order to verify the robustness and reliability of the benchmark results, the following section further uses the number of green invention patent applications, the number of green utility model patent applications, and the number of granted patents to carry out the robustness test.

#### Core explanatory variable

The explanatory variable in this paper is the NUP to indicate whether the city was implemented by the New Urbanization Pilot Policy in that year, with Covered taking the value of 1 and uncovered taking the value of 0.

#### Control variables

The NUP is not an exogenous shock in the full sense of the word, so it is necessary to control further the variables that may affect the locality to become a pilot city and, at the same time, will affect the local GTI. Referring to the research of Li et al. (2023) [14], the control variables selected in this paper mainly include (1) Industrial structure. Industrial restructuring is the dynamic and effective allocation of resource elements in various regions and sectors, so the reallocation of labor is affected by industrial restructuring. We use the proportion of value added of the secondary industry to GDP to measure the industrial structure. (2) Level of economic development. The level of regional economic development reflects the local salary compensation to a certain extent, which is conducive to strengthening the attraction of high-quality labor, thus optimizing the labor structure and enhancing cities’ GTI. This paper adopts the logarithmic GDP per capita to measure the level of economic development. (3) Population density. Regional population density reflects the degree of population concentration to a certain extent, which is conducive to attracting high-skilled talents to the city, improving the quality of employees, and optimizing the labor structure. In this paper, the ratio of total population to administrative land area at the end of the year is used as a proxy variable for population density. (4) Urbanization rate. This paper uses the ratio of the city’s household population to the city’s total population as a proxy variable for the urbanization rate of the city. (5) Human capital. This paper uses the ratio of the number of students enrolled in secondary school in the city to the registered population per 100 people. (6) Industrialization level. This paper uses the ratio of the number of industrial legal units with annual primary business income of 20 million yuan and above in the city to the household population per 10,000 people as a proxy variable for the city’s industrial level.

### Data sources and descriptive statistics

Considering that most pilot cities participating in the new urbanization policy are at the city level, this paper constructs panel data for 285 prefecture-level cities in China from 2010 to 2021 after excluding regions with completely missing data. The list of pilot cities for new urbanization is taken from the website of the Central People’s Government of the People’s Republic of China; the city green patent data is based on the International Patent Classification (IPC) code for green patents issued by the World Intellectual Property Office (WIPO), and the number of green patents is retrieved in the patent database of China’s State Intellectual Property Office (SIPO) in terms of year and region. The data for other variables are obtained from the China Urban Statistics Yearbook, the Annual Report of Urban Statistics, the EPS database, and the CSMAR database. Table 1 reports the descriptive statistics of the main variables.

**Table 1:** Descriptive statistics.

Variable	Mean	Sd	min	max	N
GTI	0.010	0.040	0	0.908	3420
NUP	0.182	0.386	0	1	3420
Industrial structure	0.458	0.111	0.107	0.898	3420
Level of economic development	10.712	0.579	8.687	12.385	3420
Population density	0.381	0.256	0.031	1.506	3418
Urbanization rate	0.561	0.151	0.197	1	3420
Human capital	0.142	0.036	0.046	0.352	3419
Industrialization level	2.651	2.029	0.160	16.412	3418

## Empirical results and analysis

### Baseline regression results

Table 2 shows the results of the baseline regression. Column (1) is the regression results without control variables and fixed effects, and columns (2)-(4) are the estimation results with the addition of control variables, city fixed effects, and time fixed effects in turn, respectively. With the gradual addition of control variables and fixed effects, the variables of new urbanization policy implementation are all significant at the statistical level of 5%, and the coefficients are positive, indicating that the implementation of new urbanization policy significantly improves the level of green technological innovation in the city, and the first research hypothesis can be verified. As China’s economy gradually shifts from high-speed growth to high-quality development, the implementation of the NUP has led China towards an innovation-oriented high-quality development mode and driven the development of urban green technological innovation, thus promoting the overall green transformation of China’s economic and social development, and Hypothesis 1 has been verified.

**Table 2:** Benchmark estimation results.

Variables	(1)	(2)	(3)	(4)
	GTI	GTI	GTI	GTI
NUP	0.012***	0.006**	0.007***	0.005**
	(5.194)	(2.521)	(3.116)	(2.093)
Industrial structure		-0.013**	-0.018**	0.019*
		(-2.124)	(-2.257)	(1.899)
Level of economic development		0.014***	0.016***	0.005
		(3.090)	(3.040)	(1.290)
Population density		0.005	0.007*	0.007**
		(1.541)	(1.874)	(1.989)
Urbanization rate		-0.013	-0.045*	-0.069**
		(-0.951)	(-1.750)	(-2.063)
Human capital		-0.011	0.010	0.030
		(-0.484)	(0.512)	(1.177)
Industrialization level		-0.002***	-0.003***	-0.003***
		(-2.635)	(-3.049)	(-2.802)
Constant	0.008***	-0.117***	-0.123***	-0.025
	(3.893)	(-3.680)	(-3.316)	(-0.705)
City FE	NO	NO	YES	YES
Time FE	NO	NO	NO	YES
*N*	3420	3415	3415	3415
*R* ^2^	0.012	0.078	0.096	0.128

Note: Values in parentheses are clustered robust t-statistics at the city level. (2)

***、**、 and * represent statistical significance at the 1%, 5%, and 10% level, respectively.

### Robustness tests

#### Parallel trend test

**Fig 3:**
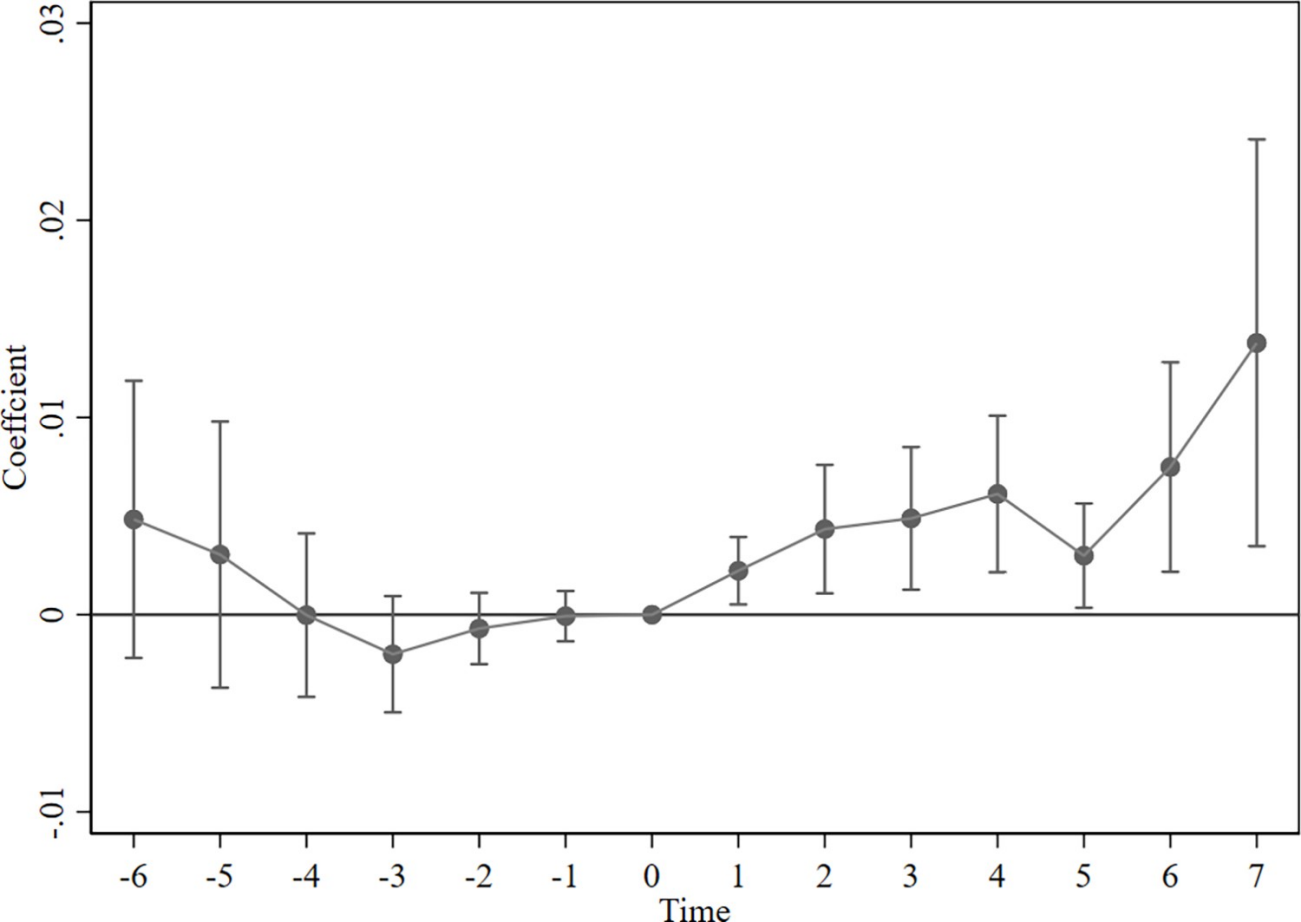
Parallel trend test.

The prerequisite for using the multi-period difference-in-difference model is to satisfy the parallel trend test, i.e., there needs to be a consistent trend in the level of green technological innovation in the experimental group and the control group prior to the implementation of the NUP. This paper draws on the study of Beck et al. (2010) [37] to conduct the parallel trend test. According to Fig 3, it can be seen that the regression coefficients from the first year to the first five years before the implementation of the NUP are not significant, but the level of GTI in the city starts to grow rapidly after the policy implementation. The Parallel trend test results indicate no significant difference in the trend of green innovation level between the experimental group and the control group before the policy implementation, and the model above satisfies the parallel trend assumption.

### Replacement of the explanatory variables

In this paper, we further use the number of green utility patent authorizations (GTI_1), green invention patent applications (GTI_2), and green utility patent applications (GTI_3) as proxy variables for the level of GTI. The regression results are shown in columns (1)-(3) in Table 3. According to the estimation results, it can be seen that the estimated coefficients of the interaction term D of the pilot new urbanization policy are all positive and significant at the 5% level, which indicates that the incentive effect of the pilot new urbanization policy on the city’s green technological innovation does not depend on the way of measurement of the explanatory variables.

**Table 3:** The robustness test of sample adjustments.

	Replacement of the explanatory variables			Adjustments to the samples	
Variables	(1)	(2)	(3)	(4)	(5)
	GTI_1	GTI_2	GTI_3	GTI	GTI
NUP	0.018**	0.021**	0.017**	0.007***	0.002***
	(2.192)	(2.020)	(2.140)	(3.539)	(3.900)
Constant	0.131	-0.208	-0.053	-0.024	-0.002
	(0.862)	(-1.464)	(-0.438)	(-0.794)	(-0.172)
CVs	YES	YES	YES	YES	YES
City FE	YES	YES	YES	YES	YES
Time FE	YES	YES	YES	YES	YES
*N*	3406	3410	3410	2616	3248
*R* ^2^	0.215	0.163	0.222	0.192	0.285

Note: Values in parentheses are clustered robust t-statistics at the city level. (2)

***、**、 and * represent statistical significance at the 1%, 5%, and 10% level, respectively.

#### Adjustments to the study sample

The choice of sample cities affects the regression results of multi-period difference-in-difference model. First, municipalities directly under the central government belong to provincial administrative units, which are directly under the administration of the central government, while prefectural-level cities are generally under the jurisdiction of the provinces to which they belong. Significant differences exist between municipalities and prefectural cities regarding authority, urbanization level, administrative level, public resources, scale of innovative talents, and so forth. Therefore, this paper tries to delete all the samples of municipalities and keep only the samples of prefectural cities. Second, the implementation area of the new urbanization policy contains multiple administrative levels such as provinces, cities, and counties, and there is a situation where some pilots are within some control group cities. Since the samples in this paper are all at the city level, only the areas where the NUP is implemented in the city will be classified as the treatment group, while the cities where there are pilots within them will be classified as the control group, which will result in bias in the estimation results. Therefore, we remove the sample of cities where county pilots are located. The regression results are shown in Column (4) of Table 3, and the interaction term of NUP is significantly positive at the 1% level.

#### Excluding extreme values from interference

A few cities may have far greater GTI than others due to resource endowment advantages or regional industrial and talent agglomeration, or some cities have far fewer carbon emissions than others due to geographic conditions, location conditions, or industrial structure. In order to avoid the interference of these two types of a few cities, the explanatory variables are subjected to the upper and lower 5% shrinkage before the regression. The results are shown in Column (5) of Table 3.

#### Placebo test

**Fig 4:**
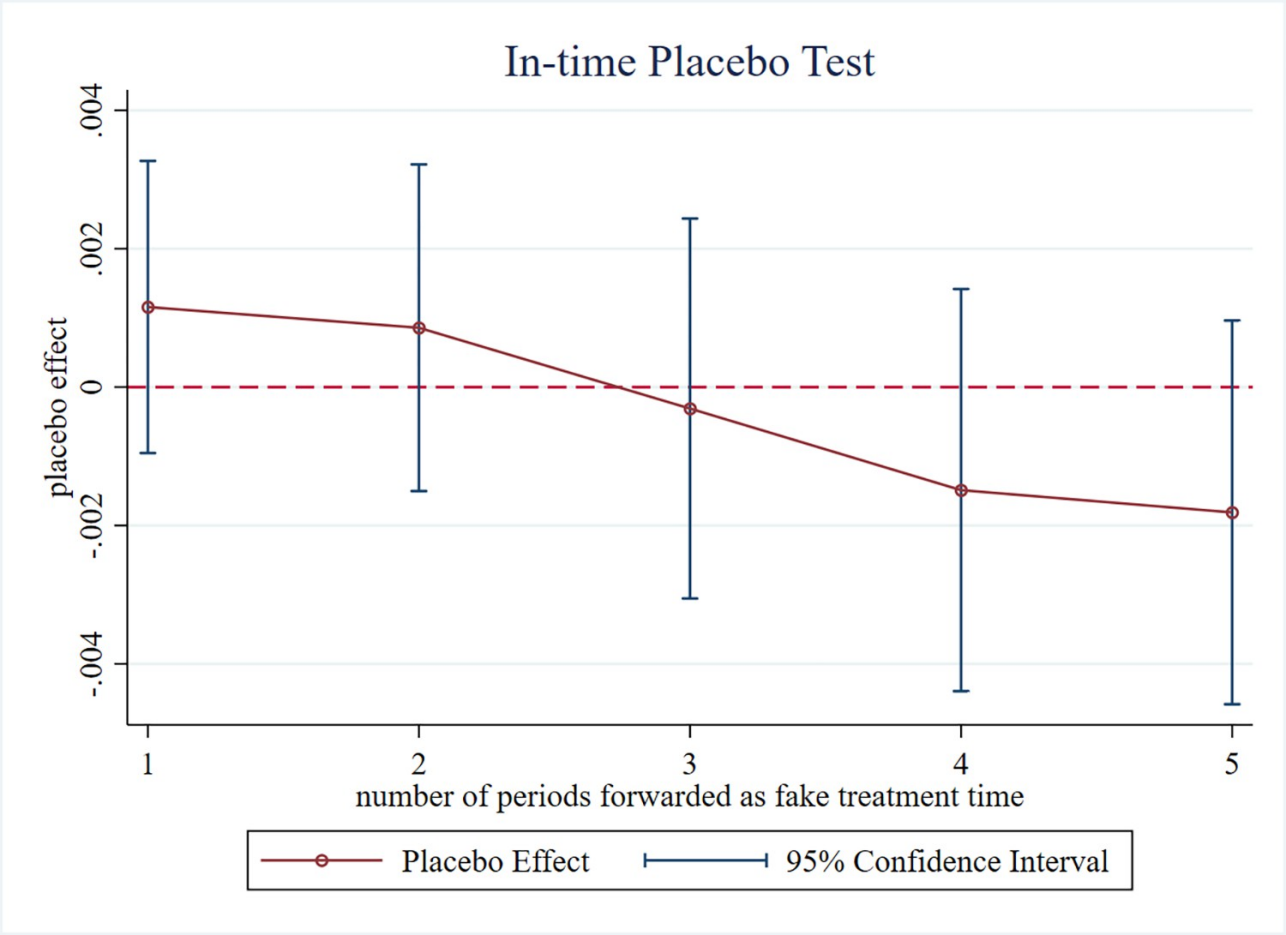
Results of the in-time placebo test.

In order to further exclude the influence of other unknown factors on the selection of pilot areas, this paper respectively advances the implementation of the NUP by 1 to 5 years to conduct a placebo test, which indicates that the basic test is robust if no significant change in the level of urban green technological innovation is observed. Fig 4 shows that none of the regression coefficients of the policy dummy variable D are significant after advancing the policy, and the coefficients are quite different relative to the baseline regression results, indicating that there are no other qualities of the cities undergoing new urbanization pilots that can lead to the development of the level of green technological innovation in the cities. The results of this paper are relatively robust.

#### Heterogeneous treatment effects

Recently, a growing number of studies have doubted the validity of the multi-period difference-in-difference model estimator with a two-way fixed effects framework due to the possibility of heterogeneous treatment effects (HTE) (Zhou et al. 2023) [38]. Goodman-Bacon (2021) proposed that the total average treatment effect in a multi-period difference-in-difference model is a weighting of the average treatment effect resulting from different samples acting as a control group [39]. This paper relies on this approach for decomposition. Since there are no individuals who always receive a treatment effect, treatment effects are categorized into three groups. Table 4 shows the result of bacon decomposition. Almost all of the weight (more than 95%) comes from the "treated vs. never treated" group, with only 2.8% of the estimated coefficients coming from the "post-treated vs. first-treated" group. Concerning the specific estimation results in this paper, the classical two-way fixed-effects framework is not seriously affected by the HTE, suggesting that the benchmark regression is robust.

**Table 4:** Goodman-Bacon decomposition.

DD Comparison	Weight	DD Estimation
Earlier T vs. Later	0.018	0.004
Later T vs. Earlier	0.028	-0.006
T vs. Never treated	0.954	0.006

In addition, in order to further address the heterogeneity treatment effect problem and the statistical bias problem of the traditional two-way fixed-effects model, we refer to the modified method proposed by Callaway and Sant’Anna (2021) as a complementary and robust test for the bias that may be caused by the traditional multi-period double-difference approach [40]. The results of the examination, shown in Table 5, are consistent with the findings of the baseline regression, further illustrating the robustness of the paper’s conclusions.

**Table 5:** Robust estimators under heterogeneous treatment effects.

	Coef.	Std. Err.	z	[95% Confidence interval]	
CAverage	0.00307*	0.00161	-1.911	-0.00008	0.00622
T2014	0.00039	0.00049	-0.807	-0.00056	0.00135
T2015	0.00225**	0.00099	-2.279	0.00032	0.00418
T2016	0.00283	0.00181	-1.569	-0.00071	0.00637
T2017	0.00339*	0.00191	-1.778	-0.00035	0.00713
T2018	0.00428**	0.00207	-2.070	0.00023	0.00833
T2019	0.00253**	0.00117	-2.165	0.00024	0.00482
T2020	0.00338	0.00236	-1.435	-0.00124	0.00800
T2021	0.00552	0.00421	-1.311	-0.00273	0.01378

#### Omitted variable bias

Considering the existence of some potential factors affecting causality, we conduct robustness tests by adding control variables to avoid estimation bias. Based on this, this paper further adds the following three variables to the baseline regression gradually: (1) Consumption market, measured by total retail sales of consumer goods (natural logarithm); (2) Infrastructure development, measured by per capita investment in fixed assets in urban municipal utilities construction completed (natural logarithm); (3) Public service, measured by the number of urban basic medical insurance participants as a proportion of the resident population. the estimation results after adding control variables are shown in columns (1)- (3) of Table 6. shows that the effect of NUP on GTI remains significantly positive, making the conclusions of this paper more robust.

**Table 6:** Avoid missing critical variables.

	Adding control variables			Control of other pilot policies			
	(1)	(2)	(3)	(4)	(5)		(6)
NUP	0.005**	0.005**	0.005**	0.006***		0.006**	0.006**
	(2.085)	(2.086)	(2.062)	(2.722)		(2.492)	(2.447)
Consumption market	0.001	0.001	0.001				
	(1.061)	(1.072)	(0.947)				
Infrastructure development		0.000	0.000*				
		(1.207)	(1.934)				
Public service			0.014***				
			(2.832)				
National big data comprehensive pilot				0.010**		0.011**	0.011**
				(2.014)		(2.104)	(2.112)
Low-carbon city pilot						0.008***	0.008***
						(2.855)	(2.824)
Smart city pilot							0.000
							(0.175)
Constant	-0.034	-0.031	-0.011	-0.036		-0.032	-0.031
	(-0.996)	(-0.928)	(-0.303)	(-1.043)		(-0.936)	(-0.944)
CVs	YES	YES	YES	YES		YES	YES
City FE	YES	YES	YES	YES		YES	YES
Time FE	YES	YES	YES	YES		YES	YES
*N*	3417	3413	3092	3417		3417	3417
*R* ^2^	0.128	0.128	0.122	0.145		0.159	0.159

Note: Values in parentheses are clustered robust t-statistics at the city level. (2)

***、**、 and * represent statistical significance at the 1%, 5%, and 10% level, respectively.

The previous section has verified that the implementation of NUP has a positive effect on city’s GTI, but since China has introduced a number of policies related to digital economy and environmental regulation during the sample period, whether these policies will interfere with the regression results still needs to be further verified. For this reason, we select three pilot policies: "national-level big data comprehensive pilot zone pilot ", "low-carbon city pilot" and "smart city pilot". The three pilot policies are gradually added into the benchmark regression in the form of control variables. The results are shown in columns (4)-(6) of Table 6. The regression results are still significantly positive after controlling for the above policy interferences, indicating that NUP does have the effect of increasing the city’s GTI.

## Further analysis

### Heterogeneity analysis

#### Differences in types of cities resources

One of the core objectives of NUP is to enhance the innovative and sustainable development capacity of cities. Resource and non-resource cities face different pressures in the development process of green technological innovation due to differences in resource types, resource endowment, and degree of utilization, and the effectiveness of the implementation of the NUP may be significantly different. The National Sustainable Development Plan for Resource-based Cities (2013–2020) issued by The State Council of the PRC classifies cities into resource growth, maturity, decline, and regeneration. In order to verify whether the NUP has the same effect on driving the development of GTI in resource-based and non-resource-based cities, this paper divides the 285 sample cities into 114 resource-based cities and 171 non-resource-based cities and performs a group regression. The regression results of columns (1) and (2) in Table 7 show that the NUP can have a significant impact on both types of cities, but the estimated coefficient on the level of green base innovation in non-resource cities is four times higher than that in resource cities. The possible reason for this is that the economic development of resource-based cities depends on local natural resources, especially the extraction of traditional energy sources such as coal, oil, and natural gas. Restricted by factors such as material base and technological capacity, the development base of green technological innovation in resource cities is relatively weak, and the promotion effect of new urbanization policies is limited.

**Table 7:** City resource and pollution Heterogeneity.

Variables	(1)	(2)	(3)	(4)
	Non-resource cities	Resource cities	Higher pollution level	Lower pollution level
did	0.008**	0.002***	-0.003	0.009***
	(2.062)	(2.855)	(-1.261)	(2.968)
Constant	0.036	0.000	-0.110*	0.038
	(0.573)	(0.004)	(-1.786)	(0.673)
CVs	YES	YES	YES	YES
City FE	YES	YES	YES	YES
Time FE	YES	YES	YES	YES
N	2049	1368	1701	1716
R2	0.176	0.277	0.059	0.276

Note: Values in parentheses are clustered robust t-statistics at the city level.

***、**、 and * represent statistical significance at the 1%, 5%, and 10% level, respectively.

#### Heterogeneity of cities pollution levels

In order to verify the Heterogeneity of the impact of new urbanization policies on GTI in cities with different pollution levels, this paper further uses the ratio of total urban CO2 emissions to GDP to represent the pollution level of cities. We divide the sample into two groups of higher and lower urban pollution levels according to the median of the ratio of total urban CO2 emissions to GDP and perform group regression. The results of Column (3) and Column (4) in Table 7 show that the NUP can significantly promote GTI when the level of cities’ pollution is low, while the policy effect is not significant when the level of urban pollution is high. The possible reason is that as China’s economy shifts from the stage of high-speed growth to the stage of high-quality development, the people’s demand for a beautiful ecological environment is growing, and environmental quality is increasingly becoming an important influencing factor for the flow and agglomeration of capital, talents, and other factors [41]. The impact of air pollution on urban development is mainly manifested in the reduction of urban quality, which in turn brings about the spatial "voting with feet" of human capital [42], and the new urbanization policy can further strengthen the concentration of human capital in the region, and increase the level of urban green technological innovation.

#### Heterogeneity in city size

In this paper, the city size is divided into small, medium and large cities according to the population, taking the number of residents population in the city in the initial year 2010 as the baseline. Cities with a population size of less than 3 million people are defined as small cities, cities with a population size from 3 million to 5 million people are defined as medium-sized cities, and cities with a population size greater than 5 million are defined as large cities. Columns (1) to (3) in Table 8 reflect the heterogeneous impacts of the NUP on GTI among different city sizes. From the estimation results, it is clear that the new urbanization pilot can significantly increase the level of GTI in cities of medium population size. The new type of urbanization is a process in which non-agricultural industries cluster in towns, and the rural population concentrates in towns. Large cities have specific scale effects and can provide perfect medical, education, judicial, and other services to absorb a large amount of labor and capital, and such cities are not the focus areas of the NUP. As for small-scale cities, the weak industrial base and limited supply of essential public services will result in the outflow of population from the region, and the promotion of GTI in the NUP will be limited.

**Table 8:** City size and locational Heterogeneity.

	City scale			City location		
Variables	(1)	(2)	(3)	(4)	(5)	(6)
	Large	Medium	Small	East	Central	West
did	0.009	0.004***	0.001	0.006	0.005**	0.004
	(1.355)	(3.015)	(1.397)	(0.968)	(2.615)	(0.919)
Constant	0.232*	0.004	-0.002	-0.021	-0.044	-0.021
	(1.912)	(0.309)	(-0.613)	(-0.202)	(-1.438)	(-0.902)
CVs	YES	YES	YES	YES	YES	YES
City FE	YES	YES	YES	YES	YES	YES
Time FE	YES	YES	YES	YES	YES	YES
*N*	1078	1019	1320	1198	1200	1019
*R* ^2^	0.291	0.365	0.198	0.197	0.209	0.122

Note: Values in parentheses are clustered robust t-statistics at the city level.

***、**、 and * represent statistical significance at the 1%, 5%, and 10% level, respectively.

#### Heterogeneity in city location

China has a vast territory and a complex topographic and geomorphologic structure, and the level of economic development and the starting point of development vary significantly among regions. Therefore, this paper divides the whole sample into three parts: east, central, and west regions to examine the heterogeneous impact of new urbanization policies on GTI under different geographical locations. The regression results are shown in columns (4) to (6) of Table 8. It can be seen that the NUP has a significant effect on the improvement of GTI level only in the central region, while it is not significant for both the eastern and western regions. The possible reason is that implementing environmental protection policies and green development strategies is stricter and earlier in the eastern region, and as the marginal effect of green technology on urban green development decreases, the dependence of cities’ GTI on external policy shocks is reduced. In addition, along with the rapid development of tertiary industry in the eastern region, the central region has gradually become the main destination of industrial transfer in the eastern region. Compared with the western region, the central region can receive the industrial transfer from the eastern region and the spillover effect of science and technology and talents, and the new urbanization policy has a more significant role in promoting green technological innovation.

### Moderating effects test

The previous multi-period difference-in-difference model estimation results and a series of robustness tests confirm that the NUP can significantly increase the level of GTI in cities, so is this effect moderated by other variables? To answer this question requires further analysis of its moderating effect. The theoretical analysis and exploratory hypotheses in the third part of the paper have already drawn the theoretical hypothesis that the NUP can enhance the level of green technological innovation would be affected by the government’s attention to environmental governance, the government’s expenditure on science and technology, and the agglomeration of scientific and technological and educational talents. In this part, this paper will be empirically tested.

#### Model construction

In this paper, we refer to Shi and Li (2020) [43] and place the moderating variables in a benchmark regression model to examine whether the moderating effects are significant. The specific model design is as Eq (2):

$$y_{it}=\alpha +\beta_{1}NUP_{it}\times M_{it}+\beta_{2}NUP_{it}+\beta_{3}M_{it}+\gamma X_{it}+u_{i}+v_{t}+\varepsilon_{it}$$ (2)

y represents the level of GTI in the city; M represents the moderating variable, which in this case refers to the government’s attention to the environment, the government’s investment in science and technology, and the agglomeration of scientific, technological, and educational talents; the main concern is the significance of the coefficients of the interaction term, *NUP*_*it*_×*M*_*it*_, and the rest of the model setup is consistent with Eq (1).

#### Moderating variable definitions

(1) Government attention: The variable definition of government attention mainly refers to the study of Bao and Liu (2022) [44], in which the sum of the word frequencies of 16 environment-related words in the government work report is counted as the degree of government environmental attention by text analysis method. In this paper, the total number of word frequencies obtained is divided by 10 to facilitate the interpretation of the estimated coefficients. The specific words and their total number of occurrences are shown in Table 9. The government work report can visually present the allocation of decision makers’ attention in a specific period, and by analyzing the local government work report, we can understand the degree of local government’s attention to ecological environmental protection and the degree of inclination to technological development. (2) Financial support: The financial support is determined by the ratio of the city government’s scientific expenditure to the total financial expenditure, which can represent to a certain extent the government’s financial support to technological innovation. (3) Talent Gathering: Talent Gathering is measured by the total number of employees in the city’s information transmission, computer services, and software industry, scientific research and technological services, geological exploration industry, and education industry, reflecting the scale of a city’s talents and the degree of concentration.

**Table 9:** Keywords selected in this study.

	Keywords in English	Word frequency
环境保护	Environment protects	4510
环保	The abbreviation of environment protects	10481
环境	Environment	34453
绿色	Green	17241
低碳	Low-carbon	2869
减排	Reduce emissions	8305
生态	Ecology	44114
环境质量	Environmental quality	1613
空气质量	Air quality	3472
污染	Pollution	9493
二氧化硫	Sulfur dioxide	1059
大气污染	Atmospheric pollution	2034
排放	Emissions	3108
能耗	Energy consumption	4022
新能源	New Energy	7291
蓝天	Blue sky	1833

#### Results of moderating effects test

Table 10 lists the results of the verification of the interaction effect. It can be found that the promotion effect of NUP on the level of GTI is significantly affected by the degree of government participation and the scale effect of talent. The government’s attention to the environment, the government’s investment in science and technology, and the concentration of talents in the city can significantly increase the level of GTI in the city. The government’s environmental attention and government investment in innovation largely reflect the government’s investment in environmental protection and innovation, implying that the implementation of the NUP has produced a noticeable "Porter effect", which significantly improves green technological innovation through the government’s environmental regulation and the increase in investment in innovation. This finding also confirms the view that government behavior has a substantial impact on regional GTI [45] and that the scale effect generated by the agglomeration of technological talents is conducive to the enhancement of GTI in cities [46, 47], and H2 and H3 can be verified.

**Table 10:** Test results of moderating effects.

Variables	(1)	(2)	(3)
	GTI	GTI	GTI
NUP	-0.001	-0.007**	-0.005**
	(-0.362)	(-2.186)	(-2.152)
Government attention *NUP	0.001**		
	(2.063)		
Government attention	-0.001**		
	(-2.515)		
Financial support *NUP		0.465***	
		(3.445)	
Financial support		0.057	
		(0.755)	
Talent Gathering*NUP			0.110**
			(2.357)
Talent Gathering			0.086
			(1.070)
Constant	-0.021	-0.005	-0.056*
	(-0.596)	(-0.153)	(-1.917)
CVs	YES	YES	YES
City FE	YES	YES	YES
Time FE	YES	YES	YES
*N*	3254	3417	2810
*R* ^2^	0.139	0.171	0.218

Note: Values in parentheses are clustered robust t-statistics at the city level. (2)

***、**、 and * represent statistical significance at the 1%, 5%, and 10% level, respectively.

## Conclusion

In this study, the establishment of new urbanization pilot areas is used as a proposed natural experiment, and based on the balanced panel data of 285 cities in China from 2010 to 2021, the multi-period DID is used to assess whether the new NUP can increase the level of green technological innovation in the cities, and thus promote the sustainable development of the cities and achieve the goal of high-quality development of China’s economy. The study results show that the NUP can increase the level of GTI in cities to a certain extent, which still holds after a series of robustness tests, such as the parallel trend test and the placebo test. Second, there is significant regional and urban Heterogeneity in the impact of new urbanization pilot policies on urban green technological innovation, and the increase is more significant for non-resource cities, low-pollution areas, central regions, and cities with medium population size. Third, through moderating effect analysis, this paper finds that the NUP significantly improves the level of GTI, this impact receives a moderating effect from the government’s attention to the environment, the government’s investment in innovation, and the agglomeration of urban innovation talents.

The research in this paper extends the existing theoretical research on urban green technology innovation. From the perspective of the study on the driving factors of urban green technology innovation, the existing research mainly focuses on the influence of the external environment such as external environmental regulation, government policy support, economic growth pressure and other external environments on urban green technology innovation, and seriously neglects the driving effect of green technology innovation driven by the pilot policy in the institutional environment as well as the internal sustainable orientation of the city. From the perspective evaluating the utility of the pilot urbanization policies, most studies have looked at the income, consumption, and industrial upgrading effects of population mobility and infrastructure development brought about by the pilot urbanization policies, but there has been less discussion of the environmental pressures brought about by the new urbanization policies. The pilot policy of new urbanization is proposed in the process of China’s rural-to-urban population transfer and the transformation of high-speed growth to high-quality sustainable growth. Considering the environmental promotion effect and the upgrading effect of science and technology brought about by the pilot new urbanization, local governments as well as scholars should be concerned.

Based on China’s urbanization process that has lasted for decades, this study provides a new research perspective on the quality of China’s urbanization on urban green technological innovation from the perspective of new urbanization pilot policies, and further provides a new theoretical framework for systematically promoting the implementation of green technological innovation strategies in cities in terms of governmental environmental regulation, governmental R&D support, and human capital agglomeration, and provides new evidence for the policy-driven behavior of countries or regions in transition.

## Policy implications and future research

Based on the empirical results of this paper and the above conclusions, this paper proposes the following policy recommendations:

It is necessary to gradually promote and expand the pilot scope of NUP and give full play to the radiation-driven and demonstration-led effect of the NUP on the development of talent concentration, low-carbon cities, and green innovation. The research in this paper shows that there is significant regional Heterogeneity in the impact of NUP on GTI in cities, and the impact on low-pollution areas, central areas, medium population size areas, and non-resource cities is more significant. Based on this, the strategy of expanding the scope of the pilot should be more orderly and prudent, taking into full consideration the actual situation of each region to ensure that the promotion of the new urbanization pilot has a more significant effect. At present, the institutional mechanism for the integrated development of China’s urban agglomerations is still unsound, the coordination of the development of large, medium-sized, and small cities is insufficient, the scale of mega-cities is expanding too fast, and some small and medium-sized cities and towns are facing a reduction in their economic and population scales. It is necessary to accelerate the construction of new urbanization, change the development mode of urbanization, and form a new development mode with city clusters as the main spatial carrier and the coordinated development of large, medium-sized, and small cities and small towns. While attracting talent, technology, capital, and other factors to gather in cities, cities should emphasize the driving and feeding of small towns and rural areas to promote better upgrading GTI in cities.

The government should lead in the construction of new urbanization, which requires the intervention of "active government". In maintaining the market economy-led construction of new urbanization, the government should take into account all factors, carry out top-level design, optimize industrial policy, solve livelihood problems, promote the total welfare of the society and the improvement of the economic level, and guarantee the efficiency and fairness, to achieve the high-quality economic growth. Specifically, when formulating new urbanization policies, the government should focus on attracting high-quality talent in the region by expanding investment in public infrastructure, promoting basic public services such as education, medical care, housing, and other public livelihood construction, and keeping high-quality talents in the region by improving the social security systems such as pension insurance, medical insurance, unemployment insurance, housing fund, et cetera, narrowing the income disparity, perfecting the assistance system and optimizing the regional social security system to retain high-quality talents in the region.

In addition, when formulating environmental policies, the government needs to realize that the improvement of the urban ecological environment is a long-term process, which not only requires strict environmental regulations but also needs to be based on the corresponding financial support for enterprises’ GTI, and to stimulate enterprises to carry out GTI through joint rewards and penalties. For enterprises in areas with high environmental regulation intensity, the government can appropriately increase the strength of research and development subsidies to mobilize enterprises to carry out substantive green innovation; for enterprises in areas with low environmental regulation intensity, the government should strengthen the eligibility of enterprises for research and development subsidies and the supervision of the use of subsidies to enhance the efficiency of using the government’s green technology research and development subsidies.

Finally, the local government should establish a comprehensive and systematic green consumption concept publicity system to foster long-term sustainable consumption habits among residents. Simultaneously, enterprises should relinquish short-sighted interests and develop a long-term investment system for green technology innovation research and development, ensuring the sustained effectiveness of both major environmental improvement mechanisms. The top-level design should prioritize guiding the establishment of a regional green coordinated development system. Local governments should actively explore the creation of collaborative air pollution prevention and control mechanisms, enhancing cross-departmental and cross-regional coordination organizations to jointly plan, formulate, and implement air pollution control programs that transcend administrative boundaries to form formidable regional pollution control forces. Additionally, fostering inter-regional exchanges on green technology innovation will continue to achieve a new paradigm of locally-driven development through green technology innovation while promoting win-win cooperation in green development.

The experience of China’s New Urbanization Policies (NUP) in promoting Green Technology Innovation (GTI) within its cities offers valuable insights that could potentially be adapted and applied in other national and global contexts. By examining the successful elements of China’s NUP, we can identify key strategies and principles that may be transferable to other countries and regions aiming to enhance their own GTI and sustainable urban development. To the perspective of policy intervention and market mechanisms, the Chinese model demonstrates the effectiveness of combining targeted policy interventions with market mechanisms to stimulate GTI. Other countries could consider implementing similar dual approaches, tailoring them to their specific economic and environmental contexts. This may involve creating incentives for businesses to adopt green technologies, as well as fostering competitive markets that reward innovation and efficiency. To the perspective of government intervention, China’s NUP highlights the crucial role of government in regulating environmental impacts and providing support for GTI. Nations looking to emulate this success could establish stringent environmental regulations and back them with appropriate enforcement mechanisms. Additionally, they could allocate funds to support research and development, making it financially feasible for businesses to engage in green innovation.

The research in this paper still has certain shortcomings and needs to be deepened:

Regarding the use of data, the data used in this study are the statistics of 285 prefecture-level cities in China, which covers a limited sample range, and there are county and township-level pilot areas for new urbanization policies, so the applicability of policy experience at the prefecture-level city level to county and township areas is induced to be considered. In order to further identify the causal effects of new urbanization pilot policies on regional GTI, future research could consider using more detailed and longer time-span data to enhance the reliability of the replication of the findings.

In terms of pilot policies, this paper only examines the impact of a single policy, the pilot of new urbanization, on green technological innovation. However, China is also actively carrying out pilot policies such as the pilot of the "smart city", the pilot of the "broadband city", and the pilot of the "carbon emissions trading", et cetera, so that future research can further examine whether the NUP can be linked with other policies to promote the effect of regional integration of resources.

## Supporting information

S1 Data (XLSX)
